# L-DOPA in Parkinson’s Disease: Looking at the “False” Neurotransmitters and Their Meaning

**DOI:** 10.3390/ijms21010294

**Published:** 2019-12-31

**Authors:** Abdeslam Chagraoui, Marie Boulain, Laurent Juvin, Youssef Anouar, Grégory Barrière, Philippe De Deurwaerdère

**Affiliations:** 1 Neuronal and Neuroendocrine Differentiation and Communication Laboratory, Institute for Research and Innovation in Biomedicine of Normandy (IRIB), Normandie University, UNIROUEN, INSERM, U1239 CHU de Rouen, 76000 Rouen, France; abdeslam.chagraoui@univ-rouen.fr (A.C.); youssef.anouar@univ-rouen.fr (Y.A.); 2 Department of Medical Biochemistry, Rouen University Hospital, CHU de Rouen, 76000 Rouen, France; 3 Centre National de la Recherche Scientifique (Unité Mixte de Recherche 5287), 33076 Bordeaux CEDEX, France; marie.boulain@outlook.fr (M.B.); laurent.juvin@u-bordeaux.fr (L.J.); gregory.barriere@u-bordeaux.fr (G.B.)

**Keywords:** dopamine, serotonin, noradrenaline, trace amines, neurochemistry, dyskinesia, intracerebral microdialysis, GPR143

## Abstract

*L*-3,4-dihydroxyphenylalanine (L-DOPA) has been successfully used in the treatment of Parkinson’s disease (PD) for more than 50 years. It fulfilled the criteria to cross the blood–brain barrier and counteract the biochemical defect of dopamine (DA). It remarkably worked after some adjustments in line with the initial hypothesis, leaving a poor place to the plethora of mechanisms involving other neurotransmitters or mechanisms of action beyond newly synthesized DA itself. Yet, its mechanism of action is far from clear. It involves numerous distinct cell populations and does not mimic the mechanism of action of dopaminergic agonists. L-DOPA-derived DA is mainly released by serotonergic neurons as a false neurotransmitter, and serotonergic neurons are involved in L-DOPA-induced dyskinesia. The brain pattern and magnitude of DA extracellular levels together with this status of false neurotransmitters suggest that the striatal effects of DA via this mechanism would be minimal. Other metabolic products coming from newly formed DA or through the metabolism of L-DOPA itself could be involved. These compounds can be trace amines and derivatives. They could accumulate within the terminals of the remaining monoaminergic neurons. These “false neurotransmitters,” also known for some of them as inducing an “amphetamine-like” mechanism, could reduce the content of biogenic amines in terminals of monoaminergic neurons, thereby impairing the exocytotic process of monoamines including L-DOPA-induced DA extracellular outflow. The aim of this review is to present the mechanism of action of L-DOPA with a specific attention to “false neurotransmission.”

## 1. Introduction

Although Parkinson’s disease (PD) was originally considered a motor disorder, accumulating evidence points to cognitive dysfunctions in the disease, including neuropsychiatric symptoms [1,2,3]. PD is generally thought to be a consequence of profound loss of the dopamine (DA) neurons of the substantia nigra pars compacta (SNc) reaching the putamen. Loss of striatal DA content can occur in aged humans, but the pattern can be different compared to the situation in PD [4,5].

Historically, the origin of the loss of DA content represented a biochemical failure of DA neurons to produce and release DA in the putamen. The metabolic precursor L-3,4-dihydroxyphenylalanine (L-DOPA) could help in restoring the biochemical activity of sick DA neurons. The clear relationship between the loss of DA tissue content and the destruction of DA neurons coming from the SNc (decrease in pigmented cells in the SNc) came later [6]. The neuropharmacological question behind the good therapeutic response of patients to L-DOPA is to understand how the metabolic precursor can work when an extensive part of the factory is dead even in de novo patients [7]. It is indeed reported that de novo patients have an approximately 70% decrease in striatal DA content and more than 50% of striatal DA fibers. L-DOPA is a serendipitous success and its mechanism is still puzzling [8].

The disease is much more complex as all monoaminergic systems are affected by the disease, although to a more variable degree across patients for the noradrenaline (NA) and serotonin (5-hydroxytryptamine, 5-HT) systems compared to the nigrostriatal DA system [5,9,10,11]. These systems have to be taken into account in the mechanism of action of L-DOPA. Indeed, a growing number of biochemical and pharmacological studies show that current medications and surgical interventions dramatically involve the 5-HT and NA system in their efficacy and/or side effects [1,9,12]. In terms of L-DOPA biochemical effects, these two systems can participate in the ability of L-DOPA to increase DA extracellular levels, raising the idea of “false neurotransmission.”

**Figure 1:**
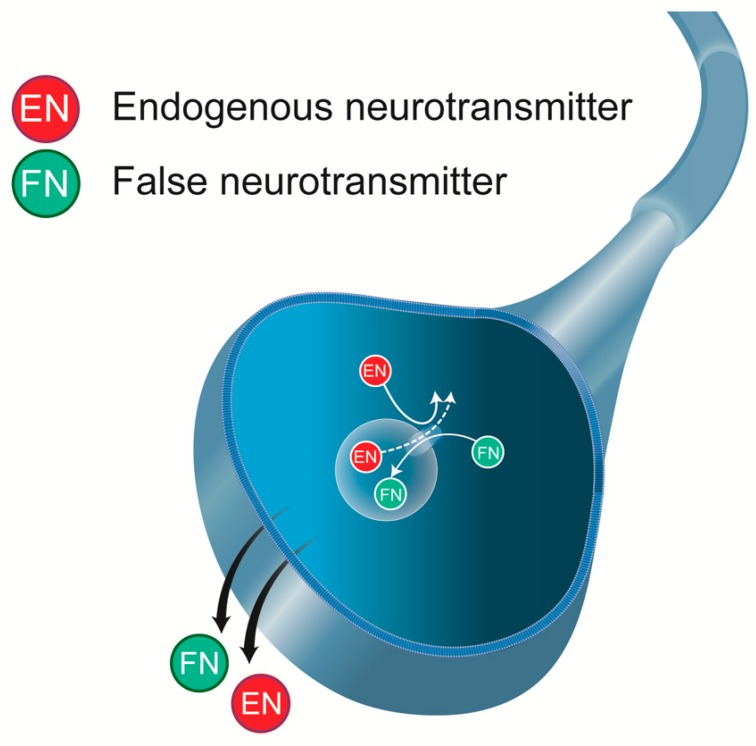
False neurotransmitter (FN). This ectopic compound, considered “unwanted,” enters a neuron or is directly formed in the neuron. The FN will replace the endogenous neurotransmitter (EN) in synaptic storage vesicles and can be released in response to a depolarizing stimulus.

**Figure 2:**
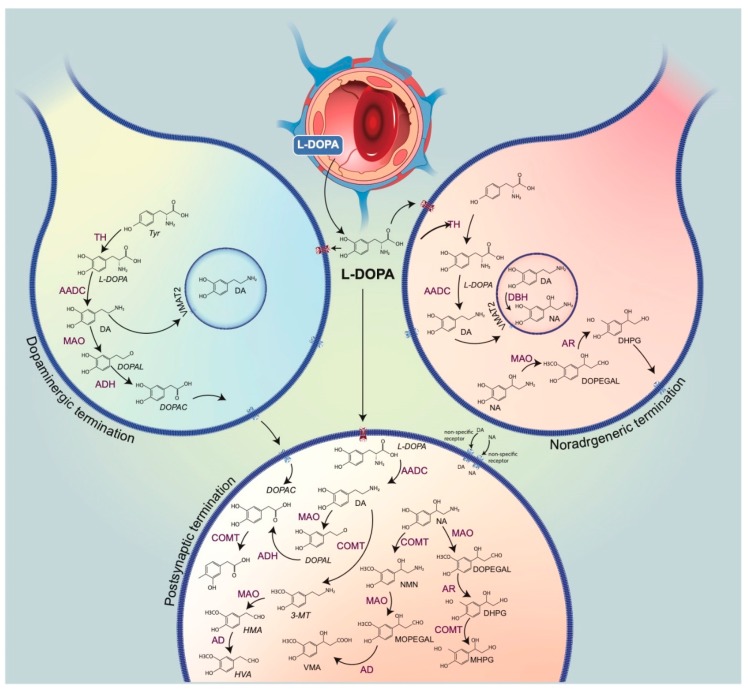
Metabolic pathways of catecholaminergic neurons. This drawing represents terminals of the dopamine (DA) neuron (left) and noradrenaline (NA) (right), a capillary, and a post-synaptic cell. L-3,4-dihydroxyphenylalanine (L-DOPA), normally synthesized from the amino acid tyrosine through hydroxylation by the enzyme tyrosine hydroxylase (the rate-limiting enzyme of catecholamine biosynthesis), can virtually reach all cells upon its exogenous administration. Neurons expressing the L-aromatic acid decarboxylase (AADC) can convert L-DOPA into DA. In monoaminergic cells, vesicular monoamine transporter VMAT2 allows for the penetration of newly synthesized DA inside the vesicles of exocytosis. In NA terminals, DA is converted by dopamine ß-hydroxylase (DBH) to NA, mainly in vesicles. DA undergoes deamination, by monoamine oxidase (MAO), to form 3,4-dihydrophenylacetaldehyde (DOPAL), which is subsequently oxidized to 3,4-dihydroxyphenylacetic acid (DOPAC) by aldehyde dehydrogenase (AD). DOPAC leaves the dopaminergic cells and is then taken up by the postsynaptic cells. DOPAC is O-methylated by catechol-*O*-methyltransferase (COMT, not present in catecholaminergic neurons), generating homovanillic acid (HVA), the final metabolite. A similar circuit occurs with NA catabolism. NA can be converted into 3,4-methoxyphenylacetaldehyde (DOPEGAL) as a result of the action of MAO. DOPEGAL, which is reduced by aldehyde reductase (AR) to 3,4-dihydroxyphenylglycol (DHPG), is released out from the NA terminals. The final product of the NA metabolism is VMA, produced by post-synaptic cells. The post-synaptic pathways can be directly alimented by the nonspecific uptake of DA and NA or, upon L-DOPA administration, by the production of DA directly in the post-synaptic cells.

We review the mechanism of action of L-DOPA with the specific angle of “false neurotransmission” it induces. “False neurotransmitter” implies the presence of an ectopic (unwanted) neurotransmitter in a neuron, which replaces the normal neurotransmitter in storage vesicles, thereby being possibly released by this neuron upon stimulation (Figure 1). It would be the case of L-DOPA-induced DA extracellular levels from 5-HT neurons [13,14]. The functional consequences of this kind of release are not clear and have been the object of several debates since the beginning of the 60s. In fact, the metabolism of L-DOPA and its products DA and NA is complex (Figure 2), with the increase in several distinct chemical species that could directly impact transmission (the trace amines for instance) or impair the normal activity of the remaining monoaminergic systems as “false neurotransmitter.”

## 2. DA Is a False Neurotransmitter in the Mechanism of Action of L-DOPA

In this section, we address the nature and the origin of DA released by the systemic injection of L-DOPA. L-DOPA is usually administered with inhibitors of the aromatic amino acid decarboxylase (AADC) such as carbidopa or benserazide. These drugs poorly cross the blood–brain barrier, thereby limiting peripheral decarboxylation of L-DOPA to indirectly enhance its penetration within the central nervous system (CNS) [15,16].

### Production of DA after L-DOPA Administration

L-DOPA could work through its decarboxylation into DA in the striatum, the mainstream hypothesis that has been prevailing for decades. L-DOPA enhances DA tissue content and extracellular levels in the striatum in animal models of PD [17,18]. The biochemical effects were often studied at very high dosage in the 60s to 90s (30–200 mg/kg), but far beyond the lowest doses triggered behavioral efficacy (0.3–1 mg/kg and beyond) [19]. It has been only 15 years since the regimen of L-DOPA progressively decreased (1–20 mg/kg) to values that would be more compatible with the clinic [20].

There is a mismatch between the striatal DA dialysate content and the actual value in the tissue. Most data reporting an increase in DA release induced by L-DOPA were obtained in a rat model of PD (unilateral lesion of ascending DA neurons from the mesencephalon to the striatum using 6-hydroxydopamine). The initial studies reported an impressive amount of DA (over 40 pg/sample, approximately corresponding to a fourfold increase in physiological values) present in the dialysates after the injection of 50–100 mg/kg L-DOPA in an experimental situation where the remaining tissue content of DA was around 5–15% of the normal content [18,21]. It was assumed that this high level of response could, in part, explain the reason why the rats developed contralateral turning behavior, i.e., the increase in DA extracellular levels would be so high that it could mimic DA agonists acting on supersensitive D_2_ receptors. However, retrospectively, these extracellular levels are probably not as high, or even extremely low at lower through behaviorally efficient doses. The contralateral rotations induced by L-DOPA, mimicking the response of DA agonists but not amphetamine, and being reported at low L-DOPA doses (3 mg/kg for instance) [22], still constitute an important paradox in the field.

**Figure 3:**
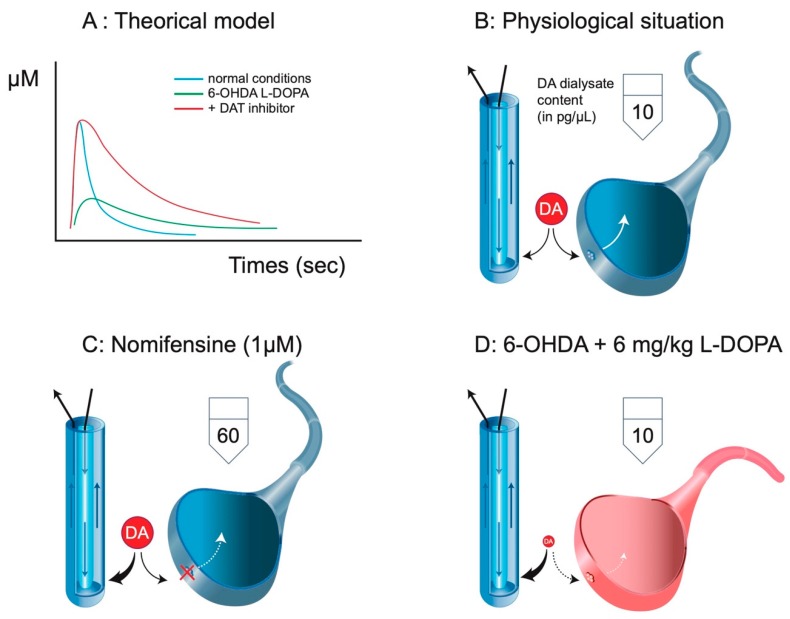
Clearance and intracerebral microdialysis. (**A**) Using fast cyclic voltammetric measurements, it is possible to illustrate the clearance of a neurotransmitter like DA. Upon stimulation, the signal (arbitrarily converted to µM) sharply reaches its maximum levels, and the clearance starts. It results in a rapid disappearance of the neurotransmitter from the extracellular space. In the presence of a DA transporter (DAT) blocker, the magnitude of the signal is not necessarily increased, but the rate of disappearance is dramatically reduced. In 6-OHDA conditions, the magnitude of the signal, if any, is lower, but the rate of disappearance is extremely low (no DAT). (**B**) Striatal DA extracellular levels correspond to a balance between the endogenous clearance and the exogenous clearance due to the microdialysis probe. In classic microdialysis experiments, the basal DA dialysate content in the striatum approximately reaches 10 pg/sample. (**C**) The local or systemic administration of a DAT blocker results in the increase in DA extracellular levels due to the loss of endogenous clearance and the increase in exogenous clearance (here, by sixfold according to [23]. (**D**) In 6-OHDA, there is no endogenous clearance of DA. The magnitude of the initial signal explaining the 10 pg obtained in the dialysate after 6–10 mg/kg L-DOPA is necessarily very low. Adapted from [20].

In 6-OHDA-treated rats, the destruction of DA fibers is massive, reaching in the case of a full lesion more than a 90% decrease in DA tissue content and DA transporter (DAT) autoradiographic binding. The loss of DAT has fundamental issues on the nature of the measured neurochemical signal using intracerebral microdialysis. The loss of clearance (longer time for DA in the extracellular space) by the reduction of DAT implies a higher rate of reuptake of DA by the microdialysis probe (Figure 3). Microdialysis converts the loss of clearance into a higher rate of collection, a reason why partial lesions of DA terminals do not have so much impact on the extracellular levels of DA collected with a microdialysis probe [18]. The system is completely modified, and we can only speculate on the meaning of these levels. In non-lesioned animals, the application of DAT inhibitors via a dialysis probe or by systemic injection strongly enhances the DA dialysate content by 5–10 fold [23,24,25]. In 6-OHDA rats, a DAT inhibitor (at least systemic injection) has no effect on basal and L-DOPA-stimulated extracellular DA levels [26]. The absence of the DAT likely implies that DA uptake by the microdialysis probe is amplified by at least 5 to 10. Therefore, the 10 pg of DA collected in a dialysate from an intact animal cannot be compared to the 10 pg of DA in a dialysate that is measured after 6–10 mg/kg L-DOPA in 6-OHDA lesioned rats. It should correspond to a 5-to-10-times lower initial output of DA from neurons (presumably 5-HT neurons, see below). In the initial description of the effect of L-DOPA and amphetamine on the rotation behavior, the authors first noticed that a dose of 20 mg/kg was required to reach normal levels of DA (as those caught in intact striatum), and second, that the rotating behavior paralleled DA extracellular levels in the case of amphetamine but not in the case of L-DOPA [18,27]. The functional value of striatal DA extracellular levels in 6-OHDA rats receiving L-DOPA is extremely poor and does not predict behavioral effects [20] (see below).

Beyond the problem related to the estimation of the actual values of DA with the microdialysis probe, it is likely that the L-DOPA-stimulated striatal extracellular level of DA does not involve the remaining striatal DA terminal fibers. As explained elsewhere [28], the extracellular levels of DA enhanced by L-DOPA in 6-OHDA rats are insensitive to drugs that normally alter the activity of DA neurons. In particular, the stimulation of D_2_ receptors by quinpirole, which is still powerful to inhibit the low basal extracellular levels of DA collected in the striatum of 6-OHDA rats, does not alter L-DOPA-induced DA extracellular levels [29]. It is likely that the vesicles of exocytosis in functional/non-functional synapses of DA neurons [30] contain higher concentrations of DA after L-DOPA injection [31], but the enhanced extracellular levels of DA, maybe in regions like the SN [32,33], could limit the excitability of DA neurons [34] and impair depolarization-dependent exocytosis from DA neurons. To the best of our knowledge, we have no evidence that the remaining DA neurons in fully lesioned rats participate in L-DOPA-derived extracellular DA in the striatum, even after the co-injection of the antagonist haloperidol (which should have relieved DA neurons from the autoreceptor’s inhibitory action), as if the released extracellular DA did not reach the remaining DA terminals. If extracellular DA is involved in the benefit/side-effects of L-DOPA, it likely comes from an ectopic source, released as a false neurotransmitter.

## 3. DA Released as a False Neurotransmitter by Serotonergic Neurons

The concept of false neurotransmitters or “substitute transmitters” was proposed during the 60s to account for the increase or decrease in sympathomimetic effects of some phenylethylamine derivatives including tyramine [35]. This idea was transposed at the level of mesencephalic slices containing raphe 5-HT cell bodies in the case of the exogenous application of L-DOPA. The authors suspected that the excitatory effect of the application of 100 µM L-DOPA on [^3^H]5-HT release in the slices was due to newly synthesized DA because the effect of L-DOPA was sensitive to an inhibitor of AADC [36].

### 3.1. 5-HT Neurons Mediate L-DOPA-Induced DA Extracellular Levels

It was very difficult to approximate the participation of 5-HT neurons in the outflow of DA by 5-HT neurons using tissue measurement. At best, the data reported the need of 5-HT neurons to decarboxylate DA after moderate [22,37] but not high L-DOPA doses [17,38]. Using microdialysis, it was clearly shown that L-DOPA-derived extracellular DA required the integrity of 5-HT neuron terminals to occur [14,33]. Beyond the lesion of 5-HT neurons, the effect of L-DOPA on DA extracellular levels can be modulated by a variety of drugs or conditions decreasing (5-HT_1A_ agonists, selective serotonin reuptake inhibitors (SSRI), deep brain stimulation of the subthalamic nucleus), or enhancing (5-HT_4_ agonist) the electrical activity of 5-HT neurons. The output of DA induced by L-DOPA in the striatum is governed by the activity of 5-HT neurons [10,20,39,40,41]. It does not imply, however, that the extracellular DA released as a false neurotransmitter from 5-HT terminals is fully effective in the striatum.

The role of striatal 5-HT terminals in the effect of L-DOPA on extracellular DA levels permits the understanding of why the output of DA is low in the striatum with respect to normal values. One-tenth to one-twentieth of the normal DA innervation of the striatum is represented by 5-HT terminals [5,42], with noticeable inhomogeneity between territories [4]. Furthermore, the firing rate of 5-HT neurons is lower than that of DA neurons (around 1 Hz for 5-HT neurons and 4.5 Hz for DA neurons) [43,44]. Finally, L-DOPA does not alter the electrical activity of 5-HT neurons either after acute or chronic injection [44,45,46]. Thus, the outflow of DA induced by L-DOPA from 5-HT terminals is likely minimal, and it is artificially amplified by the loss of clearance when addressed using microdialysis. It is noteworthy that at behaviorally relevant doses such as 1, 2, or 3 mg/kg [19,22,47], the increase in DA outflow induced by L-DOPA is very low [20,33,47,48], far from reaching physiological values in the striatum. This is consistent with the data obtained in humans where the displacement of the binding of the D_2_/D_3_ ligand ^11^C-raclopride by L-DOPA occurs, suggesting an increase in DA extracellular levels, but at levels below the expected values of physiological DA transmission [49,50]. 

### 3.2. L-DOPA-Derived DA from 5-HT Neurons at the Expense of 5-HT Function

Newly synthesized DA is released by 5-HT neurons from L-DOPA as a false neurotransmitter and has several consequences. First, in the cytosol of 5-HT neurons, it competes and substitutes the natural neurotransmitter 5-HT to reach the vesicles of exocytosis through the vesicular monoamine transporter VMAT2. The action of false neurotransmitters occurs at the expense of the normal function of the true neurotransmitter. In the case of L-DOPA and 5-HT neurons, the extracellular levels of 5-HT seemed maintained [32,46,51] or slightly decreased in a region-dependent and dose-dependent manner [51,52]. However, the nature of the extracellular levels of 5-HT measured after L-DOPA administration is different because the impulse-dependent release modulated by classical 5-HT treatments is slightly impaired by L-DOPA [44,52]. It is likely due to the newly synthesized DA chasing 5-HT from its storage vesicles [45,52,53] as suggested in the early days [13,36,54]. DA and L-DOPA also substitute 5-HT or its precursor 5-hydroxytryptophan (5-HTP) in a variety of mechanisms inside 5-HT neurons, altering the metabolic activities of 5-HT neurons [20,45]. The second consequence is that the output of newly synthesized DA occurs in all CNS areas innervated by 5-HT neurons, i.e., the entire CNS [33]. At variance with the striatum, the extracellular signal of DA measured by microdialysis is less biased in 6-OHDA rats compared to normal rats because the reuptake of DA poorly involves the DAT in extrastriatal regions. Thus, the lesion of DA neurons has poor influence on the natural clearance of extracellular DA in brain regions like the cortex or the hippocampus, which is, in part, due to the norepinephrine transporter (NET) [55]. Meanwhile, the inhibitors of the NET desipramine and reboxetine amplified by 2–3 fold the extracellular levels of DA induced by 12 mg/kg L-DOPA in extrastriatal regions but not in the striatum (too low levels of NET) [56]. In the presence of these inhibitors, the output of DA was similar in the striatum and extrastriatal region. It has been recently suggested, based on a long-term treatment of L-DOPA and the measurement of tissue markers of DA and metabolites in the striatum and the SN, that the SNr could be more involved in the motor effects of L-DOPA [57]. It had been proposed earlier as well [58], and it probably involves other key regions of the brain [59].

### 3.3. DA as a False Neurotransmitter from 5-HT Neurons: Functional Impact

L-DOPA triggers a variety of behaviors in humans and in animal models of PD [1], reproducing in the latter the benefit and side effects of L-DOPA. Does striatal DA released as a false neurotransmitter from 5-HT neurons play a role in L-DOPA-induced dyskinesia? We do not know. The main evidence that 5-HT neurons are involved in L-DOPA-induced dyskinesia comes from the seminal article by Carta et al. [60]. Briefly, the authors reported that a massive lesion of 5-HT using 5,7-dihydroxytryptamine impaired the ability of L-DOPA to induce dyskinesia in 6-OHDA rats having presented L-DOPA-induced dyskinesia before the 5-HT lesion [60]. The authors went further by showing that 5-HT_1A_ agonists known to reduce the activity of DRN 5-HT neurons and the impulse-dependent release of 5-HT also inhibited L-DOPA-induced dyskinesia. These findings were reproduced in 1-methyl-4-phenyl-1,2,3,6-tetrahydropyridine (MPTP)-treated monkeys receiving 5-HT_1A_ agonists and/or bearing a partial lesion of 5-HT neurons [61,62,63]. Since then, numerous data have confirmed that the reduction of 5-HT neuron activity is one of the best strategies to date to limit L-DOPA-induced dyskinesia in the animal model of PD (here are some reviews on the subject: [1,20,62,64,65,66,67]). 

All the data support the involvement of 5-HT neurons in L-DOPA-induced dyskinesia, but none of them support a role for striatal DA released by striatal 5-HT terminals as a false neurotransmitter. It has been reported that the expression of D_2_ receptors specifically in 5-HT neurons of the DRN reduced and even suppressed L-DOPA-induced dyskinesia [46]. These data elegantly confirm that silencing 5-HT neurons of the DRN dramatically limits the occurrence of L-DOPA-induced dyskinesia. In sharp contrast with the behavioral analysis, the authors report a modest decrease of 12 mg/kg L-DOPA-induced extracellular levels in the group expressing D_2_ receptors in DRN 5-HT neurons compared to controls. They evoked the possibility that the lower increase in extracellular DA induced by L-DOPA in the D_2_ receptor-expressing rats could be under a threshold of DA levels producing dyskinesia but sufficient to permit the motor benefit. The argument is based on the seminal article [60]. Yet, the difference between the two groups in the study of Sellnow et al. (2019) [46] is too low to conclude because the initial release of L-DOPA-derived DA from 5-HT terminals would not be so different, and the small difference could have been amplified 5–10 fold due to the absence of clearance of DA in the striatum (see above). Second, the idea of threshold is dangerous for a “false neurotransmitter” [68] especially if we consider that (1) L-DOPA-induced dyskinesia occurs at doses of L-DOPA 4 to 6 times lower than those used by the authors, thereby at DA extracellular levels far below the levels reported at 12 mg/kg L-DOPA in D_2_ receptor-expressing animals, and (2) an L-DOPA-induced raise in extracellular levels of DA could also involve non-exocytotic or impulse flow-independent processes [53,69]. The data by Sellnow et al. (2019) [46] would thus confirm that part of L-DOPA-derived DA from 5-HT neurons is insensitive to 5-HT neuron electrical activity. Finally, L-DOPA can produce dyskinesia independently from a raise in striatal DA extracellular levels in MPTP-treated monkeys [70]. Thus, there is no clear evidence that striatal DA released by 5-HT neurons in the striatum plays a role in L-DOPA-induced dyskinesia in rats [48], monkeys [70], and humans [71].

While the hypothesis of the increase in striatal DA transmission induced by L-DOPA has been driving research in the field of dyskinesia for years, it is clear from animal studies that extracellular quantities of striatal DA can be extremely low during dyskinesia, too low to imagine an excess of striatal DA transmission. The levels of DA released by L-DOPA could also go toward a progressive decrease after chronic treatment with L-DOPA in rats [51,72]. The opposite way of understanding L-DOPA-induced dyskinesia could be that the striatal level of DA is too low to maintain proper filtering of the numerous incoming information from multiple brain regions, all of them receiving 5-HT innervation and L-DOPA-derived DA.

The extrastriatal effects of L-DOPA via DA are probably underrated, although, again, it is released as a false neurotransmitter and the direct link between this type of release and an action on neighboring cells is difficult to establish (see below). At least, in the experiment proposed by Sellnow et al. (2019) [46], the authors report good evidence that the extracellular levels of DA could have an inhibitory feedback on ectopic D_2_ receptors expressed by DRN 5-HT neurons by reducing the electrical activity of DRN 5-HT neurons. This effect would occur in the DRN, and not the striatum. Beyond this, it is unclear whether the effects reported in the striatum by L-DOPA are related to striatal DA or to other mechanisms. It has been reported that L-DOPA in 6-OHDA rats can induced a variety of cellular effects including an enhancement of c-Fos expression at high doses [73], and low doses [74], and which depend on the integrity [73] and/or the activity of 5-HT neurons [75]. Similarly, other striatal factors are altered after moderate doses of L-DOPA producing dyskinesia [75,76,77]. While these effects are interpreted as a consequence of the increase in striatal L-DOPA-derived extracellular DA, one cannot exclude the participation of incoming neuronal systems from the cortex, the thalamus, or the midbrain to cite a few [78,79]. Changes in expression of neuronal markers of activity can also be observed in conditions of low DA tone, as suggested by the increase in c-Fos expression induced by antipsychotic drugs [80,81,82]. L-DOPA favors a low DA tone in the striatum compared to physiological values and a high DA tone in other brain regions [59]. This imbalance could be at the origin of the specific effects reported at the level of the striatum.

### 3.4. Conclusion

The release of DA as a false neurotransmitter from 5-HT neurons is a complex situation particularly in the striatum where DA is supposed to act, but it is released at ectopic sites and not necessarily close to DA receptors, and at low quantity compared to the physiological situation. The role of striatal DA in the benefit of L-DOPA is also questionable for the same reasons [20]. It could be interesting to determine the contribution of striatal DA released by 5-HT neurons in the overall effect of L-DOPA, if any.

## 4. DA Released as a False Neurotransmitter by Noradrenergic Neurons

### 4.1. Endogenous DA Can Be Released by NA at Low Levels

Several data suggest that DA can be released by noradrenergic fibers, particularly in the cortex or the hippocampus [83,84,85], the latter region being very poorly innervated by DA neurons. Being the precursor of NA, and having a better ability to cross the vesicular membrane through VMAT2 compared to NA [20], DA is likely present in the vesicles of exocytosis in NA neurons [86]. Nevertheless, one particularity of NA neurons is the presence of DA-β-hydroxylase (DBH) at high concentrations inside the vesicles of exocytosis. DBH is also present in the cytosol but has a lower influence on the NA content of the vesicles of exocytosis. Thus, an output of L-DOPA-derived DA from NA terminals would not totally be a false neurotransmission and could even be expected. However, when L-DOPA was administered in 6-OHDA rats, the signal on DA release disappeared when 5-HT neurons were destroyed [33], suggesting that the contribution of NA fibers in L-DOPA-derived DA outflow is negligible. In line with this, the lesion of NA fibers using DSP-4 did not reduce L-DOPA-simulated extracellular levels of DA [48,56], but rather amplified them in the hippocampus and the substantia nigra [56]. Thus, there is no clear evidence indicating that NA neurons mediate the excess of DA extracellular levels induced by L-DOPA. It is noteworthy, however, that the physiological extracellular DA levels issued from NA neurons in the cortex or the hippocampus are low compared to the release of DA elicited by 3 mg/kg L-DOPA and ridiculous compared to 12 mg/kg L-DOPA. Thus, the contribution of NA neurons to release DA could be masked after L-DOPA. L-DOPA does not alter the electrical activity of locus coeruleus (LC) NA neurons [87], whereas the long-term treatment with L-DOPA does not alter LC NA neuron activity except in dyskinetic rats [88]. The output of L-DOPA-derived DA by 5-HT neurons can be potentiated by NET inhibitors, only at a moderate (12 mg/kg) but not a low (3 mg/kg) dose of L-DOPA. DA released by 5-HT neurons as a false neurotransmitter can occur nearby NA terminal fibers, a site of physiological release of DA.

### 4.2. DA as False Transmitter on NA Function

The presence of DA in excess within NA neurons probably has some consequences on NA release and function. It has been reported that 6 mg/kg L-DOPA (plus benserazide) dramatically enhanced striatal NA release in 6-OHDA dyskinetic or non-dyskinetic rats [48,89]. It had been reported earlier that a higher dose of L-DOPA (50 mg/kg) enhanced striatal NA release, an effect potentiated by iron deficiency [90]. In 6-OHDA rats, the effects of L-DOPA are massive corresponding to a more than 20-fold increase in the basal values of NA. This result suggests that NA can be synthesized from newly synthesized DA inside vesicles of exocytosis as it is often admitted. Nonetheless, this assumption is far from that to be evident. First of all, as a false neurotransmitter present in NA terminals, DA can promote the synthesis of NA and could also chase NA from its storage vesicles [35]. Other studies did report a modest twofold increase in striatal NA release (Millan, Gobert, Rivet, unpublished data) or no effect on cortical NA release upon the administration of 2.5 mg/kg L-DOPA in mice [91]. More directly, it has been reported that a large dose of L-DOPA (50 mg/kg plus 12 mg/kg carbidopa) inhibited NA release in the cortex but increased the extracellular levels of its metabolites 3,4-dihydroxyphenylglycol (DHPG) and 3-methoxy,4-hydroxyphenylglycol (MHPG) [92]. This effect was reduced by desipramine or the α_2_ adrenergic receptor antagonist yohimbine. The false neurotransmitter concept is clearly illustrated with those data where DA in excess competes with the other monoamine toward VMAT2, and even the NET, transiently increasing the concentration of NA in the cytoplasm, thereby enhancing its degradation by monoamine oxidases [93]. Newly synthesized DA promotes the leakage of NA from the vesicles to the cytosol. The high dose used in the study of Dayan and Finberg (2003) is irrelevant for the clinic, but it is a dose required to illustrate that the phenomenon of displacement from storage vesicles is possible, like the transient expulsion of 5-HT from 5-HT neurons that can be seen only at a high L-DOPA dose [53]. Meanwhile, the effect of L-DOPA on NA tissue content is rather conflicting as some studies report no effect, increase, or decrease in NA tissue content depending on the dose of L-DOPA, the brain area investigated, and/or the time of sacrifice after the injection of L-DOPA [9,12]. There are likely opposite mechanisms operating on NA terminals governing the outcome of NA outflow after L-DOPA.

**Figure 4:**
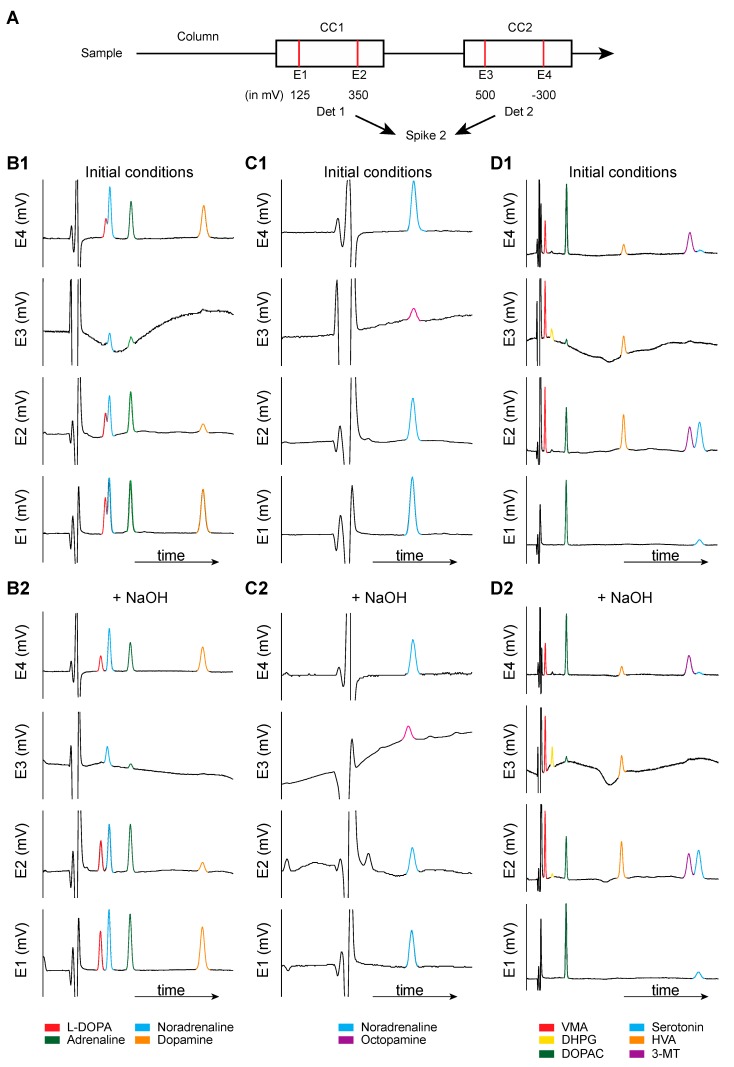
Chromatographic conditions for monoamines measurement: Focus on NA. The purpose of these chromatograms is to illustrate that the measurement of NA using classical procedures, which is not an easy task, because several species can be eluted at the same time. (**A**) Experimental procedures. We used classical procedures for the chromatographic conditions, as recently published [94], with some modifications as regards to the electrochemical detection. A conditioning cell (5020, ESA) was placed before the manual injector and set at +450 mV. Then, after the column, two coulometric cells (5011, ESA; two electrodes each) were placed in series, each cell being connected to one coulometric detector (CoulochemII, ESA). The potential at the four electrodes was as follows: +125, +350, +500, and −300 mV (reduction). The detectors were connected to the CED 1401 interface (Cambridge Electronic Design Ltd.), and signals were acquired using the spike 2 software (Cambridge Electronic Design Ltd.). B. Chromatograms (mV as a function of time, in minutes) obtained for each electrode for different groups of molecules: First column (**B1**,**B2**): L-DOPA, NA, adrenaline, DA; second column (**C1**,**C2**): NA and octopamine; third column (**D1**,**D2**): VMA, DHPG, DOPAC, HVA, 3-MT, and 5-hydroxytryptamine (5-HT). It is possible to act on the time of elution of the compounds by modifying the pH (here by adding 20 µL NaOH, the below panels B2, C2, and D2), the concentration of pairing ions, and the initial concentration of methanol (here 7%). Note that L-DOPA and NA are confounded in the initial condition and can be separated by basifying the mobile phase. Nonetheless, it is convenient to have different potentials here because some compounds do not generate signals at low potentials (E1), including octopamine, DHPG, and 3-MT. Other compounds would have given signals in these conditions: MHPG, 5-hydroxyindole acetic acid, 5-hydroxytryptophan, tryptophan, and most likely the various metabolites of L-DOPA.

A last technical point should be considered when studying the effect of L-DOPA on NA release in 6-OHDA rats. NA is often, if not, exclusively measured through high-pressure liquid chromatography coupled to electrochemical detection. NA is normally eluted in the solvent front so that high concentrations of pairing ions are used to delay the elution of NA. Using this procedure, an acceptable separation from the solvent front can be obtained, enabling the high increase in gain required for the electrochemical detection of low concentrations of NA. Numerous compounds coming from the metabolic pathways illustrated in Figure 2 can be eluted in standard chromatographic procedures and produce an electrochemical signal (Figure 3). Additionally, L-DOPA can be eluted close to NA, and its potential of oxidation is similar or even below that of NA. In the Figure 4, we report in-house chromatograms generated using two coulometric cells in series set at four different potentials for illustrating the technical and chromatographic challenge of measuring NA levels. In conditions with high concentrations of sodium octyl sulfonate (SOS, 200 mg/L mobile phase), a first run with standards solutions showed that L-DOPA and NA were almost confounded while adrenaline and DA were separated. The second example shows the elution time of octopamine and NA, which was almost confounded. However, octopamine needs a higher potential to release electrons (only seen at 500 mV), and it becomes technically easier to get rid of it by working at low potentials. In the third example, the metabolite of NA DHPG was eluted at the time corresponding to L-DOPA, NA, and octopamine, but the small peak was detected at higher potentials only. The metabolites of NA and adrenaline VMA (early elution), and the metabolites of DA homovanillic acid (HVA) and 3-MT, were separated. Of note, in addition to NA/L-DOPA versus DHPG/octopamine, the potential of oxidation can allow some compounds to be discriminated such as 5-HT versus 3-MT, sometimes co-eluted (not here). In slightly basifying the mobile phase, we could obtain fair separation of the peak of L-DOPA and NA or DHPG and NA, but the pics of NA and octopamine were totally confounded. Yet, the separation between L-DOPA and NA would not be sufficient with microdialysis studies. Indeed, these are standard solutions at 100 pg/µL sample injected. Using microdialysis, the peak of NA is approximately 1000 times lower and requires a very high gain. Several researchers are using amperometric devices because the signal/noise ratio is much better, allowing neurochemists to reach a very high sensitivity. However, these systems imply the application of one potential only, which is usually set above the optimal potential for NA, and could possibly generate signals from unwanted compounds. Thus, whether or not the peak measured at the time of elution of NA actually corresponds to NA is a real issue with tissue and microdialysis experiments. Unfortunately, in the studies reporting the effect of L-DOPA on NA release, the authors did not indicate their conditions to get rid of L-DOPA and some metabolites (DHPG, octopamine, and possibly other compounds including the numerous metabolites of L-DOPA, see below) in dialysates. 

This is important because the reported results are curious. Indeed, the increase in NA release induced by L-DOPA in 6-OHDA rats was only slightly reduced or not altered in animals bearing an additional lesion of NA neurons [48,89]. At best, the release of NA is not from neuronal origin, as discussed by the authors, and could come from blood and peripheral tissues. In any case, the reported effects are likely not related to the impact of L-DOPA on striatal NA terminals. At worst, the chromatographic procedures did not permit to discriminate the peak corresponding to NA with other peaks in the chromatograms, including L-DOPA or its metabolites (see below). 

### 4.3. Overall Consequences of DA as a False Neurotransmitter in 5-HT and NA Neurons

The monoamine transporters lack a clear selectivity [95]. When L-DOPA is administered, it is likely that the monoamines in the extracellular space compete on the SERT, NET, and available DAT if present, and also contribute to a partial non-exocytotic release of DA, 5-HT, and/or NA [45,53]. As reviewed for the mechanism of action of the local application of 5-HT in the vicinity of DA terminals [96], it becomes technically difficult with microdialysis to further the mechanism of action of drugs when the activity of the transporters is modified: the consequences of impairment of the transporters displace the reuptake of the neurotransmitter toward the probe, amplifying the small effect. Other transporters are probably involved in the clearance of biogenic amines like the organic cation transporters [97]. In addition to biogenic amines, there might also be other chemical species such as trace amines and L-DOPA derivatives that compete on these transporters [98].

## 5. L-DOPA, Its Own False Neurotransmitter?

Some strong arguments support the idea that L-DOPA itself can be a neurotransmitter [99,100,101]. Numerous groups of neurons express tyrosine hydroxylase without expressing the AADC while several cells can express AADC without tyrosine hydroxylase [102,103]. L-DOPA naturally appears as a possible end product of tyrosine hydroxylase neurons without AADC. It has been reported that L-DOPA can be released in an exocytotic way upon depolarization in different tissues [104]. Other arguments suggest that L-DOPA may directly act on receptors to modulate neuronal activity. Thus, L-DOPA could act on β1 or β2 adrenergic receptors to promote NA and DA release in vitro [105]. More recently, it has been proposed that the orphan receptor GPR143 could be a receptor for endogenous L-DOPA at the level of the retina [106,107]. L-DOPA has an affinity in the micromolar range for GPR143, which promotes cytosolic Ca^2+^ enhancement upon L-DOPA stimulation in the retina [107]. GPR143 has a widespread distribution and could therefore mediate some of the actions of exogenous L-DOPA [99], at least at moderate doses. Thus, it is not a clear “false neurotransmitter” situation as L-DOPA would be one natural ligand of this receptor. 

Nonetheless, DA is also involved in this transmission and, as previously discussed, is released as a false neurotransmitter. Indeed, DA is a natural ligand devoid of efficacy at GPR143 activity (antagonist) but with a similar binding affinity compared to L-DOPA [107]. Exogenous L-DOPA would be acting as an agonist, bypassing its natural transmission, and this can participate in the benefit and side effects of L-DOPA. DA would inhibit the action of L-DOPA, and it looks like it is a matter of balance between the two compounds that can be different between regions [99]. 

## 6. A Full Family of “False Transmitters”: The Trace Amines and L-DOPA Derivatives

“There are several ways in which substitute [false] transmitters may accumulate. The best known means is to provide a precursor that will compete in normal metabolic pathways with endogenous physiological substrates” [35]. Exogenous 5-HTP enters DA neurons, which produces 5-HT competing with DA, and DA neurons release 5-HT as a false neurotransmitter [108]. The situation is similar to that described for L-DOPA. These are basic mechanisms but probably correspond to the tip knowledge of these mechanisms. Upon chronic administration, and possibly acute administration, the accumulation of L-DOPA-derived products inside monoaminergic cells should go largely beyond DA itself, and, in turn, DA, which is also produced in all cells expressing the AADC, can generate a number of products that can have a biological influence. The last point that we will address here is the possibility that the ectopic presence of DA in the cytoplasm of different cells including glial cells or 5-HT neurons could alter the activity of monoamine oxidase (MAO) (including MAOB), thereby modifying the balance of substrates like phenylethylamine, one among others trace amines that can act as a false neurotransmitter.

### 6.1. Metabolites of L-DOPA

L-DOPA is also a substrate at catechol-O-methyltransferase (COMT) leading to the formation of 3-O-methyl-DOPA (3-OMD). This pathway is targeted in clinics using the inhibitors tolcapone, entocapone or opicapone; both entocapone and opicapone limiting the peripheral disappearance of exogenous L-DOPA in the treatment of PD due to their poor CNS penetrant properties [109,110,111]. However, 3-OMD could have a biological effect when it is produced in the CNS. The intracerebroventricular injection of 3-OMD reduced DA turnover and locomotor activity while it impaired DA uptake in rat striatal membranes and PC12 cells and increased oxidative stress [112]. The behavioral and neurochemical effects of 3-OMD are still present upon sub-chronic intracerebroventricular administration [112,113]. Upon local application on mixed cultured mesencephalic neurons and striatal astrocytes, L-DOPA displayed neuroprotective effects on DA neurons via the involvement of astrocytes. The co-application of 3-OMD prevented the effect of L-DOPA [114]. The authors provided evidence that equimolar 3-OMD concentration (100 µM) impaired the uptake of L-DOPA by astrocytes. It is still unclear whether the reported effects upon intracerebroventricular administration or local application on cell cultures are effective after its endogenous production from exogenous L-DOPA. However, 3-OMD accumulates in CNS and the periphery and its half-life is much longer (15 h) than that of L-DOPA (1.30 h) [2], and the data indicate that 3-OMD competes with L-DOPA, at least on transporters. In addition, 3-OMD might not act as a “false neurotransmitter.”

Conversely, the α-methylated analogues of tyrosine or L-DOPA and, notably, α-methyl-DOPA are also possible metabolites that indirectly interfere with monoamine function. Such an action is not related to the reduction of catecholamine synthesis but likely occurs via their accumulation under distinct forms within monoaminergic neurons. α-methyl-DOPA was also given as an antihypertensive agent in humans, acting as an α2 adrenergic receptor agonist and likely mobilizing some “false neurotransmitter” actions at noradrenergic terminals [35]. The latter action is due to the degradation of α-methyl-DOPA into α-methyl-DA (a first possible false neurotransmitter) by AADC and α-methyl-NA (a second false neurotransmitter) by DBH. One may wonder if exogenous L-DOPA actually increases the concentrations of α-methyl-DOPA in the body. In one study, 80 mg/kg L-DOPA plus 20 mg/kg carbidopa was given to rats, and the plasma content of L-DOPA, 3-OMD, and α-methyl-DOPA was measured over hours using mass spectrometry [115]. Whereas L-DOPA peaked at 30 min and progressively decreased, 3-OMD and α-methyl-DOPA were detected at 15 min after L-DOPA ingestion and reached a maximum 4 and 8 h after the injection. The concentrations remained quite elevated after 24 h. Thus, α-methyl-DOPA, like 3-OMD, can accumulate in the plasma and likely in the brain. 

### 6.2. Trace Amines and Metabolism of DA

Biological effects can be triggered, at least in the striatum, by DA degradation products such as 3-MT [116]. It acts at α_2A_ receptors in the range of 0.3–3 µM (IC_50_ values) and with low affinity (over 10 µM) at D_1_, D_2_, and α_2C_ receptors [117]. It also stimulates trace amine-associated receptor 1 (TAAR1) with sub-micromolar affinity [116]. The *meta*-*O*-methyl metabolites 3-MT, normetanephrine, and metanephrine are more potent than their respective precursors DA, NE, and epinephrine at TAAR1 [118]. The data are not clear as regards to 3-MT impact on motor behavior, because its intracerebroventricular injection has been shown to reduce [119] or increase [120] locomotor activity in rodents. Recently, it has been reported that the overexpression of the human COMT in mice bearing a lesion of DA neurons and receiving chronic L-DOPA administration led to a higher susceptibility of mice to develop L-DOPA-induced dyskinesia [121]. Such an effect was associated with a higher production of 3-MT in these mice. The tissue and extracellular levels of 3-MT are dramatically enhanced by L-DOPA [57,121]. The main origin would be the striatal neurons expressing the COMT. The substrate could be directly L-DOPA-derived DA within the cell provided that the cell co-expresses the AADC. Alternatively, the extracellular levels of 3,4-dihydroxyphenylacetic (DOPAC) are elevated in L-DOPA-treated rats from sources that are not clearly identified, presumably not only 5-HT neurons [14]. DOPAC could be the main substrate for COMT to increase 3-MT content. Moreover, the ability of MAO to transform 3-MT into HVA would be reduced by chronic treatment with L-DOPA in 6-OHDA rats [122], possibly resulting in an accumulation of 3-MT.

### 6.3. The Trace Amines

Other compounds can impair the neurotransmission of classical biogenic amines. These compounds are commonly referred to as trace amines. Some trace amines are related to phenethylamines (related to catecholamines) and include phenethylamine (PEA), *N*-methylphenethylamine, phenylethanolamine, *m*-tyramine, *p*-tyramine, 3-MT, *N*-methyltyramine, *m*-octopamine, *p*-octopamine, and synephrine. Tryptamine is also considered a trace amine [98,123]. These molecules are endogenously produced by the complex metabolic pathways of phenylalanine, tyrosine, or tryptophan, initiated by the amino acid decarboxylases including AADC. Their endogenous concentration is often low and the metabolic pathways are considered minor pathways with respect to those leading to the synthesis and catabolism of the classical biogenic amines. Some trace amine concentrations are modified in the plasma of de novo Parkinson patients, whereas treated patients displayed another pattern [124,125]. Among the main differences, the authors reported an increase in tyramine levels, and a small decrease in octopamine or PEA levels compared to a control group of humans. The authors discuss this pattern as possibly reflecting an increased activity of tyrosine decarboxylase, an increased activity of MAOB activity in platelets, and a decreased activity of peripheral DBH. It is difficult to determine whether the CNS concentrations are paralleling the reported changes in the plasma. The passage of tyramine, PEA, tryptamine, and octopamine from the periphery to the CNS is unclear. They could cross the blood–brain barrier by passive diffusion [98]. On the other hand, they are also produced in the CNS. The interest for trace amines has grown recently with the discovery of the TAARs family [98]. As far as Parkinson’s disease is concerned, both TAAR1 and TAAR5 stimulation in rodents could alter motor control [116,126,127,128].

No data have specifically focused on the effect of L-DOPA on the distribution of trace amines in the brain. Of note, L-DOPA increases the activity of methionine adenosyltransferases [119]. It leads to enhanced 3,4-dimethoxyphenylethylamine concentrations and perhaps α-methyl-DOPA. After its intracerebroventricular injection in rats, 3,4-dimethoxyphenylethylamine enhanced both the binding of DA in striatal tissue and the locomotor activity [119]. Moreover, the increase in cellular DA levels in numerous cell types might occupy both MAOA and MAOB, which could alter the normal rate of degradation of some trace amines (tyramine, PEA, octopamine to cite a few). Other trace amines or L-DOPA derivatives (α-methyl-NA for instance) are poorly degraded by MAO, and their lifetime is longer compared to other trace amines.

### 6.4. Impairment of Monoaminergic Transmissions by Trace Amines

It is likely that some but not all trace amines together with the products of α-methyl-DOPA act as a “false neurotransmitter.” It has been well described on catecholaminergic transmission and the list reported by Baldessarini (1975) [35] is the following: the products of α-methyl-DOPA, α-methyl-DA, and α-methyl-NA, the product of α-methyl-tyrosine metaraminol, tyramine, and its β-hydroxylated metabolite octopamine, α-methyl-octopamine, DA, 5-HT, adrenaline, α-methyl-adrenaline, dihydroxynorephedrine, or 3,5-dihydroxy-4-dimethoxyphenylethylamine. All these compounds have been found to be taken up by monoaminergic terminals (essentially catecholaminergic), stored, and released upon depolarizing stimuli. On the other hand, the mechanisms are not totally clear. In particular, PEA is not released by depolarizing stimuli in various preparations, indicating that it is likely not stored [98]. Its β-hydroxylated analogue phenylethanolamine would be stored. Tyramine could diffuse across membranes, although it has been shown to be released in striatal slices upon veratridine but not potassium (K+) stimulation [129]. Nonetheless, tyramine acts as amphetamine to promote a non-exocytotic release of DA on virtually all in vitro (preloaded ^3^H-DA, neo-synthesized ^3^H-tyrosine, endogenous DA) and in vivo (endogenous DA) models [96]. It indicates that tyramine, like amphetamine, profoundly alters the function of storage of DA and leads to the reversal of the function of the DAT. This was similar to the release of NA at the periphery and CNS [35]. The exogenous application of 5-HT also promotes a non-exocytotic release of DA [24,96]. In the case of tyramine, it is linked to the accumulation of the amine in the terminals, and maybe not necessarily related to the massive storage inside vesicles.

The notion of “false neurotransmitter” is often linked to the release of the compound that may act on not-well-defined targets. In most cases, the above-cited compound trace amines have less affinity than those of the classical biogenic amines toward adrenergic and DA receptors. The discovery that the TAARs could be a target of trace amines leads to the following scenario: the accumulation of the trace amine inside the vesicles of exocytosis would permit to reach high synaptic concentrations upon depolarizing stimuli that would not be otherwise reached upon simple diffusion. This is still possible, but the thesis needs experimental data. There is a third possibility that is most likely. Trace amines accumulate in monoaminergic terminals. Some of them would indirectly cause leaks of the stored neurotransmitter, while others could actually be stored. In any case, the main effect of trace amines would be to reduce the available store of releasable neurotransmitter, and this would already occur at low concentrations of trace amines. The effect obtained with the lowest concentrations of amphetamine, tyramine, or even 5-HT in vitro on DA release is an inhibition of the K+ or electrically induced stimulation of DA release [96]. Without doubt, several trace amines could reach the terminals of 5-HT, NA, and DA neurons. 

No clear evidence supports this hypothesis, but we could recall some intriguing findings. The total loss of L-DOPA-induced DA release in the striatum of MPTP-treated monkeys after chronic L-DOPA injection is curious [70]. Even if the integrity of the 5-HT system was not addressed in this experiment, the lesion of 5-HT neurons induced by L-DOPA would be limited in dyskinetic monkeys [61,130]. In 6-OHDA rats, chronic administration of L-DOPA has been shown to reduce 5-HT tissue contents in the brain in some [51,131,132] but not all studies [57]. At 12 mg/kg [96], but not 3 or 6 mg/kg chronic L-DOPA [48], extracellular levels of 5-HT were reduced by half, and the ability of L-DOPA to increase extracellular levels of DA was also reduced [28,72]. Whether the reduction of L-DOPA-stimulated DA release and basal levels of 5-HT was only due to a lesion of 5-HT neurons has not been proven. The lesions of 5-HT neurons by L-DOPA have been reported but the magnitude is low [131,132]. Therefore, an accumulation of trace amines inside 5-HT neurons could also be compatible with the decreased ability of L-DOPA to enhance DA extracellular levels in the brain.

## 7. Conclusions

L-DOPA promotes false neurotransmitters, starting by its product DA, in 5-HT neurons and perhaps in NA neurons. The situation of NA release is very unclear as it is poorly related to NA neurons in some studies. Additional chromatographic controls should be performed to ensure that the electrochemical signals correspond to NA. Several chemical species can be eluted in addition to those involved in the metabolic pathways of catecholamines and indoleamines when L-DOPA is administered. Similar comments apply to the chromatographic signals for DA or 5-HT, but the constraints are less important than those for NA.

The pattern of release of DA induced by L-DOPA does not correspond to the physiological condition, and the extent to which DA released in the striatum plays a role in L-DOPA’s action needs additional studies. Some trace amines, as well as L-DOPA derivatives, have been shown to behave as false neurotransmitters, but the ultimate finding that they are released in vivo as false neurotransmitters in the CNS upon L-DOPA administration is still pending. However, before being released, these compounds could be accumulated in monoaminergic terminals. In reference to Figure 1, the presence of these chemical species inside monoaminergic terminals could reduce the vesicular content of stored monoamines. 

The amelioration of L-DOPA therapy is ultimately conditioned by a better understanding of the metabolic pathways involving monoamines, L-DOPA, and trace amines. The DA activity of L-DOPA is likely dependent on the activity of 5-HT neurons and the presence of trace amines. These two factors could be involved in on–off fluctuations and the development of dyskinesia (unpredicted loss or gain of response upon L-DOPA injection and magnification of pulsatile stimulation of DA receptors). Acknowledging that some trace amines and L-DOPA derivatives are less sensitive to MAO activity [35], the inhibition of MAO could favor the presence of biogenic amines in the vesicles of exocytosis and enhance the predictability of responses to L-DOPA. On the other hand, the increase in newly synthesized DA in the cytoplasm of cells could also lead to enhanced auto-oxidation and raise the presence of reactive oxygen species [20,133]. It is now clear that the benefits and side-effects of L-DOPA are multifactorial, and the proper determination of each component, including the direct effects of L-DOPA, is needed. These considerations might be important to better address the full spectrum of metabolic and functional responses triggered by L-DOPA in PD in order to select the best strategies to combat the numerous motor and non-motor side effects. 

## Abbreviations

AADC	aromatic *L*-amino acid decarboxylase
AD	aldehyde dehydrogenase
ADH	alcohol dehydrogenase
COMT	catechol-*O*-methyltransferase
DA	dopamine
DBH	dopamine ß-hydroxylase
DOPAC	3,4-ihydroxyphenylacetic acid
DOPAL	3,4-dihydroxyphenylacetaldehyde
DOPEGAL	3,4-methoxyphenylacetaldehyde
HVA	homovanillic acid
HMA	4-hydroxy-3-methoxyphenylacetaldehyde
L-DOPA	*L*-3,4-dihydroxyphenylalanine
MAO	monoamine oxidase
MHPG	3-methoxy-4-hydroxyphenylglycol
3-OMD	3-*O*-methyl-DOPA
MN	metanephrine
3-MT	3-methoxytyramine
MOPEGAL	3-methoxy-4-hydroxyphenylglycolaldehyde
NA	norepinephrine
NMN	normetanephrine
PD	Parkinson’s disease
PNMT	phenolethanolamine-N-methyltransferase
SNc	substantia nigra pars compacta
TH	tyrosine hydroxylase
Tyr	tyrosine
VMA	vanillylmandelic acid
